# Characterization of Multipath Effects in Indoor Positioning Systems by AoA and PoA Based on Optical Signals†

**DOI:** 10.3390/s19040917

**Published:** 2019-02-21

**Authors:** Álvaro De-La-Llana-Calvo, José-Luis Lázaro-Galilea, Alfredo Gardel-Vicente, David Rodríguez-Navarro, Ignacio Bravo-Muñoz, Felipe Espinosa-Zapata

**Affiliations:** Department of Electronics, University of Alcalá, Alcalá de Henares, 28801 Madrid, Spain; josel.lazaro@uah.es (J.-L.L.-G.); alfredo.gardel@uah.es (A.G.-V.); david.rodriguezn@uah.es (D.R.-N.); ignacio.bravo@uah.es (I.B.-M.); felipe.espinosa@uah.es (F.E.-Z.)

**Keywords:** multipath, indoor positioning, optical signal, PSD sensor, light positioning, infrared, visible light communication (VLC)

## Abstract

In this paper, we characterize and measure the effects of the errors introduced by the multipath when obtaining the position of an agent by means of Indoor Positioning Systems (IPS) based on optical signal. These effects are characterized in Local Positioning Systems (LPSs) based on two different techniques: the first one by determining the Angle of Arrival (AoA) of the infrared signal (IR) to the detector; and the second one by working with the measurement of the Phase shift of signal Arrival from the transmitter to a receiver (PoA). We present the obtained results and conclusions, which indicate that using Position Sensitive Devices (PSD) the multipath effects for AoA have little impact on the measurement, while for PoA the positioning errors are very significant, making the system useless in many cases.

## 1. Introduction

The problem of indoor location has been a subject of intense study and research in recent years. So far, proposals have been successfully developed to provide solutions to specific applications, with different degrees of accuracy and complexity. However, the challenge to achieve the implementation and applicability obtained by outdoor positioning and navigation systems remains. The final goal would be to combine both indoor and outdoor positioning systems to provide a continuous navigation solution transparent to the end user.

For some time now, it has been accepted that, in many indoor activities, both professional and private, knowing the position of the user brings an added key value that provides a new set of capabilities for a given specific application.

In indoor positioning, where the environment is complex (walls, objects, etc.), no technology prevails as global positioning system (GPS) does in outdoor positioning systems. Sometimes the target environment restricts the design to a particular Indoor Positioning System (IPS) technology, directly related with accuracy, range, or scalability [1,2,3,4,5].

The presence of IPS anywhere is a fact [6,7,8,9]. A great research effort is currently being done to develop an IPS based on several technologies, as their location data will enable numerous applications. Among the most popular applications are: pedestrian tracking [10]; location-based services [11,12] in public and commercial centers [9]; assistance services in daily activities (Ambient Assistant Living (AAL)) [13]; location and tracking of users in geriatric and hospital centers [14,15]; location and tracking of emergency intervention agents (e.g., police/firefighters) [16,17,18]; location and guidance of autonomous vehicles in industrial environments and automated car parks [19,20]; tracking of high value goods during storage; extra information for users via augmented reality [21,22]; Internet of Things (IoT) [23], etc.

Many different systems have been proposed during recent years [3,24]: based on computer vision [24], radio waves such as ultrawideband (UWB) [25] or radio frequency identification (RFID) [26], ultrasounds [27], optical signals [28], and, more recently, new approaches based on inertial measurement units (IMUs) [10,29,30] and radio frequency (RF) communications networks such as global system for mobile communications (GSM) or wireless local area network (WLAN) [31]. Some of them, such as IPS based on infrared and ultrasound, are low cost solutions, easy to deploy with high accuracy location results in a wide range of applications. These solutions are low cost because the components to develop them are low cost with minimal maintenance. Currently, there are many requirements to consider when choosing the right technology for an IPS application. As has been discussed in [1], some decision parameters are: accuracy, precision, coverage area, required infrastructure, market maturity, privacy, update rate, user interface, system integrity, robustness, availability, scalability, number of potential users, degree of intrusion, and legal coverage, etc.

Advances in technologies of materials, electronics, and communication facilitate the continuous improvement in the performance of sensorial systems. The choice of sensors clearly depends on the application and the user’s requirements. The continuous evolution of IPS can be shown through several papers aimed at reviewing the state of the art [1,2,3,4,5].

Taking into account the comparative results obtained from these references, we can state the following:

- For ultrasound based systems the positioning accuracy is around several centimeters, with a coverage restricted to several meters but with the handicap that they are very affected by multipath effects.
- Systems based on audible signals have low accuracy and low precision (meters) although they have the advantage to be extremely low cost.
- If the technology used is WIFI, Bluetooth, or ZigBee the accuracy goes from 1.5 m to several meters, is vulnerable to access point changes, and may require special user equipment, although it is low cost with a very wide coverage.
- Ultra-wideband (UWB) presents an accuracy in the range of the tens of centimeters with high precision but also with a high cost.
- The use of geomagnetic field or inertial systems provides accuracies up to 2 m, not requiring infrastructure so they have a very low cost, but in the geomagnetic field case an initial mapping is required and in the case of inertial systems the error is cumulative.
- Systems based on computer vision can have accuracies up to centimeters depending on the coverage, but are very sensitive to light conditions.

In the case of IPS based on infrared signals, the traditional systems had an accuracy in the range of tens of centimeters and even could reach meters as they were very vulnerable to the effects of sunlight and multipath. Currently, infrared systems based on Position Sensitive Devices (PSD) have been developed [28,32,33], which determine the position using Angle of Arrival (AoA) techniques providing a positioning of high accuracy and high precision (in the range of millimeters). Additionally, as it will be shown in this paper, they are practically immune to multipath effects (similar to computer vision systems).

Regardless of the technology used, the most common problems faced by IPS are related to signal quality, deployment strategy of positioning anchors (antennas, receivers, transmitters, etc.), non-line-of-sight situations, dynamic location, interference from other devices, and, similar to outdoor positioning, multipath effects [34]. Among them, multipath effects can make a significant contribution to error in many typical indoor environments, especially for technologies designed to obtain high accurate measurements [35,36,37].

Range systems based on incoherent optical signals are normally carried out by means of flight time measurements on pulsed signals [38] or phase shift measurements on continuous-wave (CW) modulated signals [39]. Phase-based techniques do not intrinsically provide any multipath mitigation technique and pulse systems, usually implemented with laser, require very high bandwidth to discriminate between multipath components near the direct line-of-sight (LOS) path. Most of the solutions proposed to reduce the multipath effects have been developed for outdoor positioning. Classical methods are based on a special correlator design [40] while more recent approaches use multipath estimation methods [41]. The latter techniques are based on the concept of multipath estimation using a delay-locked loop [42,43]. These techniques provide greater multipath rejection than classical ones. Nevertheless, they require longer integration times and higher sampling rates.

Unfortunately, none of these mitigation techniques are directly applicable to current optical systems [44]. The main constraint is determined by the strong balance between the signal to noise ratio (SNR), the field-of-view (FoV) of the optical devices, and the channel bandwidth to obtain the accuracy, coverage, and dynamic response requirements of the IPS applications [39]. Due to this compromise, the achievable channel bandwidth using low-cost upgraded devices is in the order of tens of MHz, limiting the possible range of techniques to be applied. Time discrimination methods, such as those used in the pulsed laser range, require much higher bandwidths to resolve the multipath indoors, while correlator-based methods would provide very limited mitigation considering the available bandwidth using affordable scanning systems.

In order to address the mitigation of the multipath effects, recent researches propose a new technique for the development of IPS based on optical signals [28]. Instead of measuring Time of Arrival (ToA), Phase of Arrival (PoA), or Phase Difference of Arrival (PDoA), these novel IPS systems are based on AoA measurement using PSD sensors+optics devices, which allow to work with low frequency modulations without the need for long integration times, high sampling rates or high bandwidths. For such a system, the hypothesis is that multipath effects will have little influence. Therefore, to characterize the multipath effects for these different techniques (the new one based on AoA and the previous ones based on PoA), a realistic multipath scenario based on the research carried out in [45,46] has been done.

In this paper we evaluate the multipath effect on two different IPS systems to determine the position (distance) from optical emitter, based on the same sensor (PSD). The first one performs the measurements by determining the angle of arrival and the second one the phase of arrival. To determine the multipath effect in different situations, both models will be evaluated in same conditions. The received signal is obtained from the composition of direct LOS signal and multipath components reaching a receiver, where the power and delay of each component are calculated using radiometric and geometric equations.

## 2. Background

We have been working on IPS systems development for over a decade. One of the research lines is focused on the development of IPS based on optical signals. In this line, works have been carried out based on the measurement of the signal Phase shift on its arrival at one or more detectors and on the determination of the signal angle of arrival. In addition, work has been done to model the reflections of the optical signal in indoor and to quantify and measure the signals reaching the detector due to effect of multipath.

In [32], the sources of electrical errors in a PSD sensor system and its correction are described. In [33], the geometric model of a PSD sensor-optics system and its calibration process are presented. Based on these works, we have designed a system for determining the 3D position of mobile agents using the angle of arrival (AoA). In [28] a preliminary test of the performance of an Indoor Positioning System Based on a PSD Detector has been shown.

The main drawbacks of technologies used in indoor positioning are related to multipath (MP) effects due to non-LOS signals reaching the detectors.

Looking solely at IR systems, the model of optical signal reflection reported in [45] enables to model and analyze how multipath affects AoA [47] and PoA measurement techniques. Therefore, we take this model into account in our study. In [46] we have develop a method and tool to measure the signal reaching detectors as effect of MP. In this work an initial test has been done to determine if RSS received at each electrode of the PSD sensor could be a subtractive composition of currents, which could partially compensate the MP.

In the literature there are several research works that compute the channel impulse response with recursive methods [48], multiple input multiple output (MIMO) [49], Monte Carlo simulations (MMC) [50,51,52], or combining recursive and MMC methods [53]. Many studies on calculating the channel impulse response due to the multipath of the optical signal consider surface reflection in the environment as Lambertian or Phong reflection models [54]. Channel impulse response is suitable to analyze MP effects in communications [55,56,57,58] and LPS based on PoA or ToA [35,36,59,60], but in LPS algorithms based on AoA additional information is required [46].

The algorithms described in [46] have been used to analyze the MP effects in both PoA and AoA based positioning systems. The signal that the detectors receive is formed by the contributions of the MP coming from all the reflections of the light in the environment (considering multiple rebounds). Since MP is produced by reflections on continuous surfaces (with an infinite number of points), it is required to discretize the space [46]. For this purpose, walls, floor and ceiling of the environment are divided into a grid of cells. From the energy coming from the emitter, the signal strength that reaches each of the cells is calculated. To model the reflection, each of these cells have been considered as a point emitter located in its center, which will emit according to a specific reflection model to the rest of the cells. These in turn will re-emit to the rest of cells and so on, until after the *k*th rebound they reach the detector.

**Figure 1:**
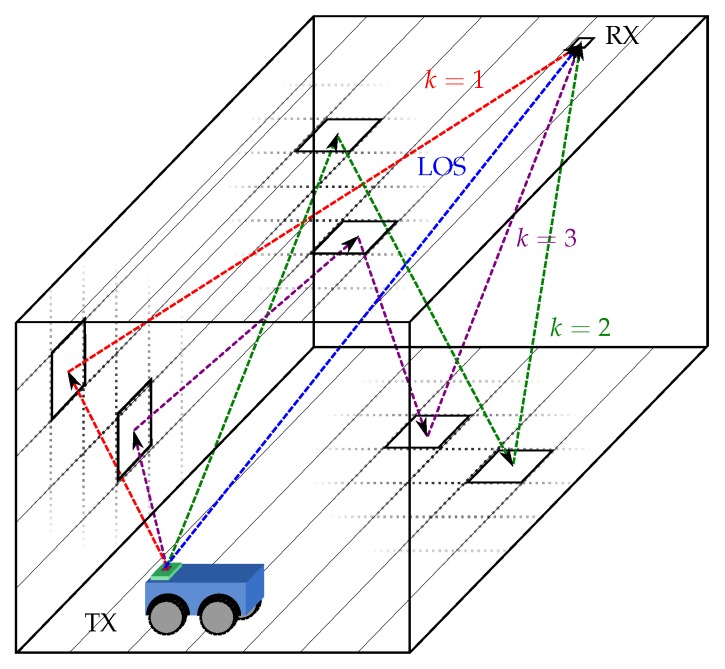
Diagram of line-of-sight (LOS) path and different rebounds. Emitter: TX; Receiver: RX.

Figure 1 presents a diagram showing 3 example situations of MP, with one, two, and three rebounds before reaching the detector. Each MP starts at the transmitter and goes to a cell in the environment, then to another cell and so on until the *k* rebound reaches the receiver.

In the case of PoA, each MP will have an associated power and phase shift that will be used to calculate the impulse response.

In the case of AoA, we get the signal strength that reaches to PSD from all the cells into which the space is divided within the detector’s FOV (PSD+optics). The signal strength that reaches each cell is calculated after considering *k* rebounds of the signal, and finally the signal strength that each MP contributes to the signal received in the detector is obtained.

The reflections of the signal in the different elements have been modeled according to the reflection model proposed in [45]. There are several state-of-art methods to model light reflection. These methods are based on physics optics [61,62,63,64], and geometrical [65,66] and empirical methods [54,67,68], but these models do not meet our requirements because they impose different restrictions and required the use of complex tools, as is described in detail in [45].

**Figure 2:**
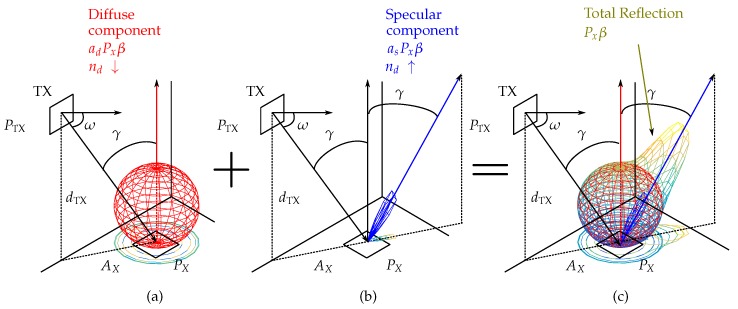
Reflection model. (**a**) Diffuse component; (**b**) specular component; (**c**) total reflection.

This model consists of two components. One component characterizes behaviors using a broad emission diagram (diffuse component), oriented according to the normal of the reflection surface. The other component characterizes behaviors using a narrower emission diagram (specular component), oriented long the direction of the beam with maximum irradiance. Figure 2 shows an example of reflection at a given point *x*. The diffuse component is shown as a sphere Figure 2a, the specular component is shown in blue Figure 2b, and the total reflection is shown in shading from blue to yellow Figure 2c.

The model characterizes the reflection of each surface in the space by mean 7 parameters $u_{as}$, $v_{as}$, $u_{nd}$, $v_{nd}$, $u_{ns}$, $v_{ns}$, β, which can be obtained experimentally from only 12 signal strength measurements, as shown [45].

### 2.1. Set-Up Definition and Coverage Modeling

The synthetic environment that has been used to carry out the characterizations is an open-plan room composed of walls, floor and ceiling. The room dimensions considered throughout the paper are 5 m × 5 m with a height of 4 m. Those values can be easily changed in the developed simulator.

The considered materials for the room surfaces are the following: the floor, terrazzo tiles, for the ceiling plaster board and for the walls a high reflection material as it could be a foam board. The parameters of the reflection model we have used for each surface of the environment are shown in Table 1. These values have been obtained experimentally according to [45].

It is worth noting that the run time of the MP determination algorithm is proportional to $n^{k}$, where *n* is the number of cells and *k* is the number of rebounds considered. In order to reduce the run times of the algorithm, we consider a cell size that depends on the rebound number. To consider 3 rebounds and a small cell size (in all of them) would imply an excessive emulation time. For example, if we consider 3 rebounds with a cell of 1×1 ${\mathrm{cm}}^{2}$, we will have approximately ${\left(1.3\times 10^{6}\right)}^{3}=2.2\times 10^{18}$ indirect paths. Programming in C using a single thread on a PC with an Intel Core i5 7500 and 32 GB DDR4-2400 RAM, the algorithm requires about $1.53\times 10^{-6}$ s to run each multipath. So the run time of this test would be of $3.366\times 10^{12}$ s. The smaller the cell size the more similar the emulated behavior is to the real behavior. In addition, the indirect paths that provide the most energy are generally those with less rebounds. Table 2 shows the cell sizes to be used in the tests and the number of indirect paths (MP) to be analyzed in the successive rebounds.

The size of the cell for rebounds 1 and 2 has been selected deliberately small in order to obtain an emulation behavior very close to the real one [46]. Emulation results have shown that using larger cell sizes could have been used without almost affecting the results obtained.

Figure 2 shows a schematic diagram with 3 different optical paths, with a different number of rebounds, one (red, *k* = 1), two (green, *k* = 2), and three rebounds (magenta, *k* = 3) until reaching the detector.

### 2.2. Emitter

In emulations, a near infrared emitter with a Lambertian emission pattern $I_{e}\left(\theta \right)=\xi \cos\theta$ has been used. Several tests have been performed in which the emitter moves along 17 positions on the room floor.

**Figure 3:**
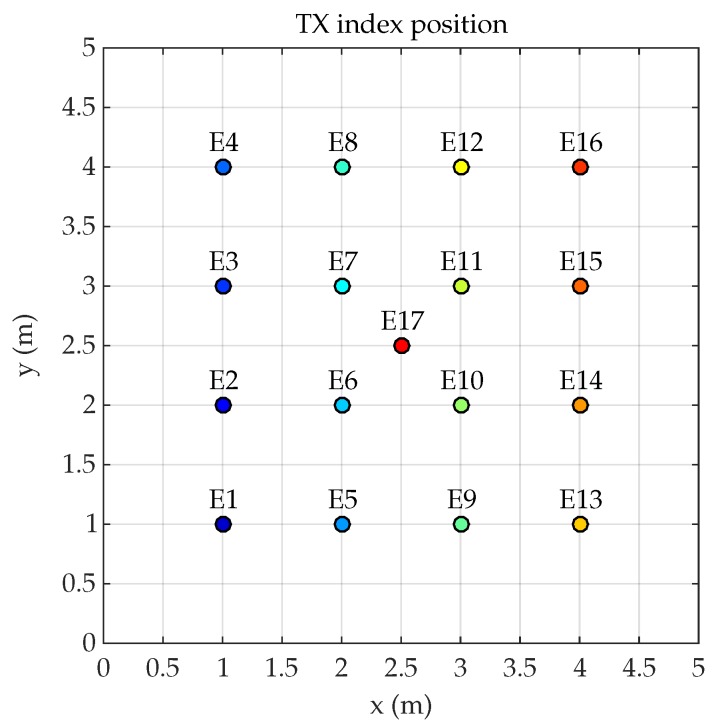
Index position of the emitter.

Figure 3 shows the emitter positions, marked with an index to identify them. The emitter shall be placed on the ground plane in all tests (plane z=0). In addition, it is always be oriented perpendicular to the ground (components of the surface vector (x=0, y=0, z=1)).

### 2.3. Receiver

**Figure 4:**
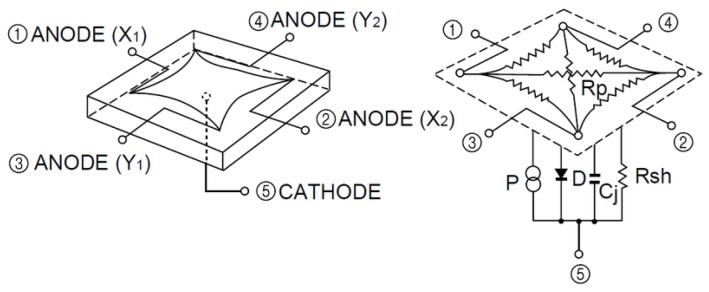
Equivalent circuit of the Position Sensitive Device (PSD) pin-cushion (image courtesy of Hamamatsu, obtained from the PSD technical information).

The synthetic detector emulates a 9×9 mm^2^ surface PSD sensor (similar to the PSD detector used later in real tests). Figure 4 shows the model of an pin-cushion two-dimensional PSD. It consists of 4 anodes and a common cathode.

The point of impact of a light beam from the emitter, collected through a lens, is determined from the output of each anode according to (1) and (2)

(1) $$\begin{array}{c} x=\frac{L_{X}}{2}\frac{\left(I_{X2}+I_{Y1}\right)-\left(I_{X1}+I_{Y2}\right)}{I_{X1}+I_{X2}+I_{Y1}+I_{Y2}}, \end{array}$$

(2) $$\begin{array}{c} y=\frac{L_{Y}}{2}\frac{\left(I_{X2}+I_{Y2}\right)-\left(I_{X1}+I_{Y1}\right)}{I_{X1}+I_{X2}+I_{Y1}+I_{Y2}}, \end{array}$$

where $I_{X1},I_{X2},I_{Y1},$ and $I_{Y2}$ are the electrical currents from the PSD sensor anode pins and $L_{X}$, y, $L_{Y}$ are the sensor dimensions.

To characterize the sensor+optical unit, the Pinhole model has been used, which allows us to obtain the geometric parameters of the system and to know the amount of energy that is collected.

**Figure 5:**
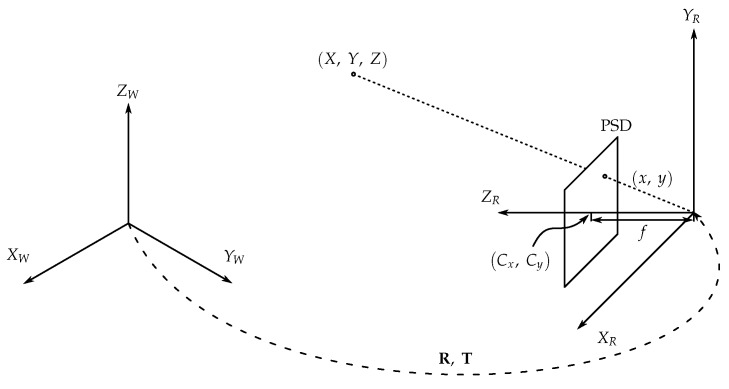
Diagram of pin-hole model.

Figure 5 shows the diagram of pin-hole model for the PSD. $\left(X_{W},\;Y_{W},\;Z_{W}\right)$ is the world’s reference system and $\left(X_{R},\;Y_{R},\;Z_{R}\right)$ the PSD sensor reference system. The point of impact in the PSD has been represented as $\left(x,\;y\right)$, the focal length of the optics as *f* and the optical center as $\left(C_{x},\;C_{y}\right)$. The rotation and translational matrices that relate the two reference systems, $\left(X_{W},\;Y_{W},\;Z_{W}\right)$ and $\left(X_{R},\;Y_{R},\;Z_{R}\right)$, are R and T respectively.

Expression (3) relates the world coordinate system with the optical coordinate system of the PSD sensor [69]

(3) $$\left(\begin{array}{c} X_{R}\\ Y_{R}\\ Z_{R} \end{array}\right)=R\left(\begin{array}{c} X_{W}\\ Y_{W}\\ Z_{W} \end{array}\right)+T,$$

where R is a 3×3 rotation matrix and T is a 3×1 translation vector.

The relationship between the optical reference system and the image is generated in the sensor image plane, according to (4)

(4) $$\left(\begin{array}{c} sx\\ sy\\ s \end{array}\right)=\left(\begin{array}{ccc} f & 0 & C_{x}\\ 0 & f & C_{y}\\ 0 & 0 & 1 \end{array}\right)\left(\begin{array}{c} X_{R}\\ Y_{R}\\ Z_{R} \end{array}\right),$$

where *s* represents the scaling factor that relates the 3D projection to the 2D projection.

The final mathematical model of the detector is shown in (5)

(5) $$\left(\begin{array}{c} sx\\ sy\\ s \end{array}\right)=\underset{A}{\underset{︸}{\left(\begin{array}{ccc} f & 0 & C_{x}\\ 0 & f & C_{y}\\ 0 & 0 & 1 \end{array}\right)}}\underset{\mathrm{RT}}{\underset{︸}{\left(\begin{array}{cccc} r_{11} & r_{12} & r_{13} & T_{x}\\ r_{21} & r_{22} & r_{23} & T_{y}\\ r_{31} & r_{32} & r_{33} & T_{z} \end{array}\right)}}\left(\begin{array}{c} X_{W}\\ Y_{W}\\ Z_{W}\\ 1 \end{array}\right),$$

where matrix A represents the intrinsic parameters which depend on the physical device and matrix RT represents the extrinsic parameters which depend on the environment geometry.

In this work, we are going to consider that there are not lens and PSD distortions, and errors are only generated by multipath effect. That is assumable because these errors can be corrected by electric and geometric calibrations. Error caused by multipath is the only error that we want to consider in this work.

**Figure 6:**
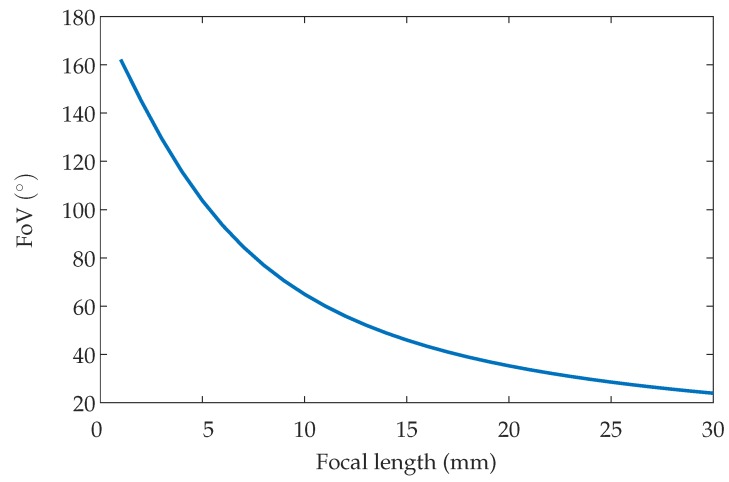
Field of view depending on focal length.

The Field of View (FoV) of the sensor can be calculated by focal length and PSD size, according to (6)

(6) $$\mathrm{FoV}=2\arctan\frac{d}{2f},$$

where *d* is the diagonal of the PSD sensor. Figure 6 shows an example of how the FoV varies depending on the focal length for the chosen PSD size (9×9 mm^2^).

**Figure 7:**
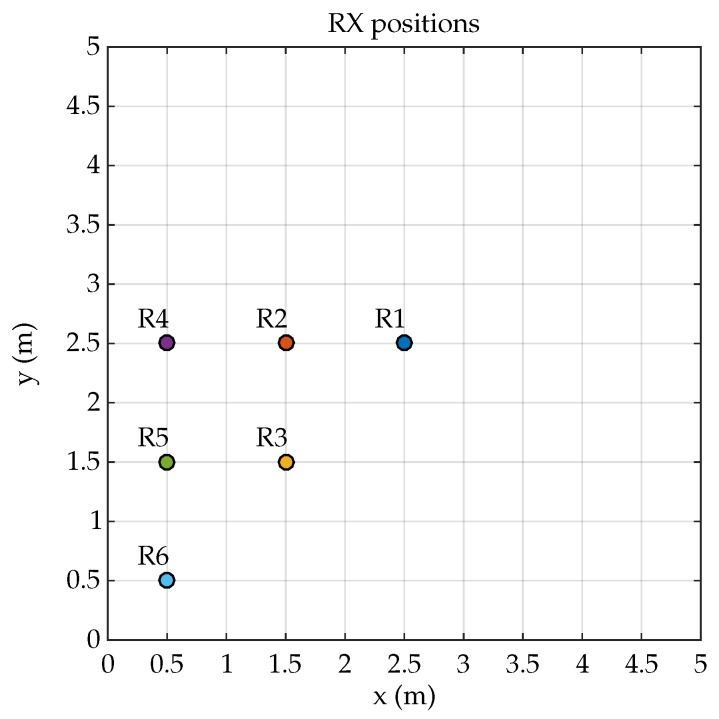
Index position of the receiver.

The detector is located in the ceiling plane (z=4 m) in different positions as shown in Figure 7. It is worth noting that with the chosen points and given that an ideal situation is emulated (with the exception of MP phenomena), the behavior in the rest of the points of the environment can be obtained by symmetry.

To check the effect of the MP, depending on the FoV of the detector, the system has been emulated considering 3 different focal lengths. Depending on the focal length, a different orientation of the detector has been considered to increase the coverage of the locations in which the emitter can be placed. The used orientations of the detector according to the focal length are shown in Table 3, expressed according to the components $\left(x,\;y,\;z\right)$ of the detector surface vector.

**Figure 8:**
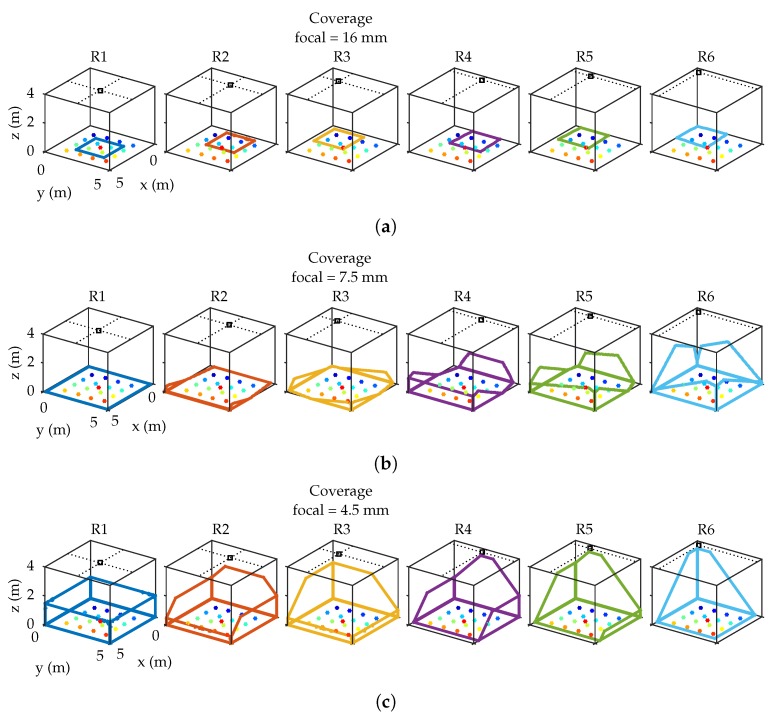
Coverage area (**a**) using 16 mm focal length; (**b**) using 7.5 mm focal length; (**c**) using 4.5 mm focal length.

Figure 8 schematically shows the coverage in the environment for each focal length value and each detector location. The thick lines identify the coverage of the detector over the room space, and dots represent the possible placement of the emitter.

For a short-focal length, the FoV of the detector covers a larger area than the area of movement for the emitter, therefore the MP are only generated from this movement area. Therefore, the smaller the FoV the less the MP affects.

To calculate the errors in AoA and PoA, the positions of the emitter and receiver are indicated in each case. Note that a single emitter and a single detector are used to determine the position by AoA and distance by PoA. The MP influence will be measured in each technique although accuracies are not directly comparable as AoA measures position and PoA distance.

## 3. Proposed Model to Calculate the Received Signal Using Angle of Arrival

To relate coordinates of the environment (3D) to the image on the PSD surface (2D), a pinhole model has been used. The algorithm presented in [46] is used to know how the indirect paths (MP) affect by calculating the signal strength with which each of the contributions reaches the detector, and obtaining, through the model, the point of the sensor where these signals reach. Thus, the center of mass of MP contributions have been obtained together with the line-of-sight (LOS) path, and currents have been weighted by the received signal strength. The mass center represents the point that would return the PSD detector.

In this particular case, the emitted signal has been a 50 kHz sinusoidal tone. We have chosen this frequency because real PSD sensor has a 150 kHz bandwidth. It has been considered that the lens and PSD do not introduce any distortions. The algorithm returns the signal strength received after *k* rebounds from the cells into which the environment is divided. Therefore, we get an array ${P_{T}}^{K}$ with as many elements as the number of cells present in the environment, where the element *i* is obtained from:

(7) $${P_{T}}_{i}^{K}=\sum_{k=1}^{K}p_{i}^{k}\;\;i=\left\{1,\;\ldots ,\;N\right\},$$

where *K* is the number of total rebounds considered, and $p^{k}$ is the array for the *k* rebound, with the successive values of the signal strength:

(8) $$p^{k}=\left[P_{1}^{k},\;\;P_{2}^{k}\;\;\cdots \;\;P_{N}^{k}\;\right],$$

where *N* is the total number of cells in the environment.

Therefore, the value of the *i* element of the $p^{k}$ array corresponds to the signal strength received by the detector from cell *i* of the environment, after *k* rebounds. In each rebound it is necessary to obtain the signal strength that reaches every cell, which in turn depends on the cell it rebounds and on the cell from the signal comes.

Once the coordinates of each cell and the model of the lens system are known, the pinhole model can be used to obtain the point on PSD reached for reflected light from each cell, as well as its center of mass, which is the equivalent point of impact. The coordinates of the center of masses, ${\vec{r}}_{\mathrm{CM}}$, are calculated according to the expression:

(9) $${\vec{r}}_{\mathrm{CM}}=\frac{P_{\mathrm{LOS}}{\vec{r}}_{\mathrm{LOS}}+\sum_{i=0}^{N}{P_{T}}_{i}^{k}{\vec{r}}_{i}^{k}}{P_{\mathrm{LOS}}+\sum_{i=0}^{N}{P_{T}}_{i}^{k}},$$

where ${\vec{r}}_{i}^{k}$ are the impact coordinates of the *i* element of the *k* rebound on the surface of the PSD, and $P_{\mathrm{LOS}}$ and ${\vec{r}}_{\mathrm{LOS}}$ are the signal strength and coordinates of the LOS component.

To calculate the position of the emitter within the environment, the positioning system proposed in [28] has been used. The following is an outline of the positioning system used: knowing the impact point on the PSD, the calibrated optical system can be used to obtain the equation of the LOS to the emitter. Knowing this line and knowing that our emitter is always move in the same plane, the position of the emitter is given by the intersection of this line with the plane.

**Figure 9:**
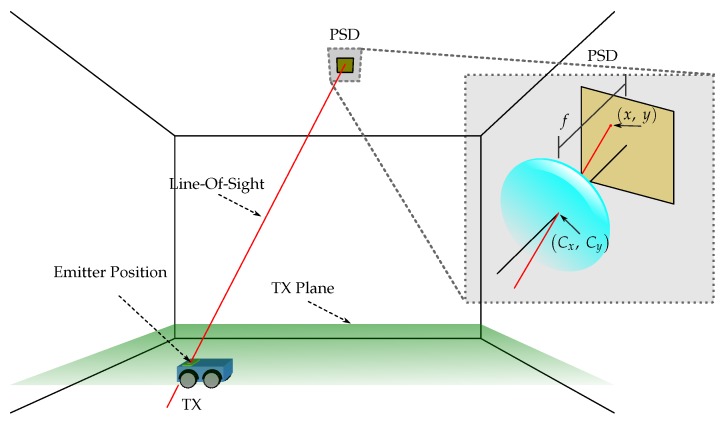
Diagram of the positioning system based on PSD sensor.

**Figure 10:**
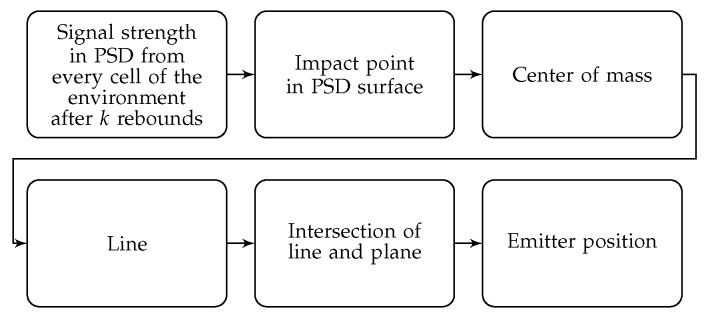
Flowchart that summarizes the steps to be taken to calculate the position of the emitter.

A diagram of the positioning system can be seen in the Figure 9 and a flowchart summarizing the steps to be taken to calculate the position of the emitter is shown in the Figure 10.

The position error is calculated from the euclidean distance between the calculated position and the current position of the emitter.

## 4. Proposed Model to Calculate the Received Signal Using Phase of Arrival

Let us consider a certain simulation environment, with known dimensions, orientation, and reflection parameters of all the surfaces in it. The impulse response h(t) from the non-LOS signals paths obtained using the algorithms given in [46] has the following equation:

(10) $$h\left(t\right)=\sum_{k=0}^{K}h^{\left(k\right)}\left(t\right),$$

where *K* is the maximum number of rebounds to be considered and $h^{\left(k\right)}\left(t\right)$ is the impulse response of the rebound *k* (please refer to [46] to see those equations in detail). The value k=0 corresponds to the impulse response of the LOS path, and values of k=1, k=2, and k=3 each correspond to the impulse response of the signals that reach the detector after 1, 2, or 3 rebounds, respectively.

Once the channel impulse response due to the MPs is obtained, the offset that the positioning system detector would measure can be calculated. The type of signal emitted and the method for the phase shift calculation will determine the delay (distance) measured between the received signal and the emitted signal.

In this particular case, the emitted signal will be a 50 kHz sinusoidal signal (to use the same frequency as in AoA). The emitter, the channel and the receiver will be considered ideal; that is, the signal received by the detectors will only be disturbed by the multipath produced by the different reflections in the environment surfaces, in the absence of any other noise. The emitter and receiver are considered perfectly synchronized and therefore the offset measured at the receiver will be directly accounted for the signal offset.

To simulate the algorithm it is necessary to discretize the time; in this case a sampling period of Ts = 0.2 ns has been chosen. The signal that the detector would receive is a sinusoidal signal of the same frequency, but with an amplitude and phase different from the emitted signal. The received signal represented with its phasor according to [70], will have the form:

(11) $$s=ℜ\left(s\right)+jℑ\left(s\right),$$

where $ℜ\left(s\right)$ and $ℑ\left(s\right)$ are the real and imaginary part of *s* respectively, and they can be obtained from the impulse response *h*:

(12) $$ℜ\left(s\right)=\sum_{i=0}^{N}h\left[i\right]\cos\left(\delta \left[i\right]\right),$$

(13) $$ℑ\left(s\right)=\sum_{i=0}^{N}h\left[i\right]\sin\left(\delta \left[i\right]\right),$$

where

(14) $$\delta \left[i\right]=\frac{2\pi t\left[i\right]}{T},$$

(15) $$t\left[i\right]=iT_{s},$$

where *T* is the period of the signal and $T_{s}$ is the sampling period.

Finally, the signal modulus and phase are given by:

(16) s=Psδs,

where $P_{s}$ is the amplitude obtained from modulus of the complex number *s*:

(17) $$P_{s}=\sqrt{{ℜ\left(s\right)}^{2}+{ℑ\left(s\right)}^{2}},$$

and the phase $\delta_{s}$:

(18) $$\delta_{s}=\arctan\frac{ℑ\left(s\right)}{ℜ\left(s\right)}.$$

The received signal captured by the detector is:

(19) $$s\left(t\right)=kP_{s}\sin\left(2\pi ft+\delta_{s}\right).$$

Knowing the value of $\delta_{s}$, the frequency of the transmitted signal, and the speed of light, the distance between emitter and receiver can be obtained using the following equation:

(20) $$d=\frac{\delta_{s}}{2\pi }Tc,$$

where *d* is the distance and *c* the speed of light.

**Figure 11:**
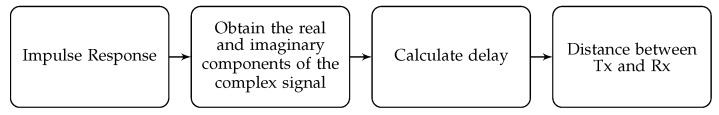
Flowchart that summarizes the steps to calculate the distance between emitter and receiver.

Figure 11 shows a flowchart with the steps required to measure the transmitter-receiver distance.

The distance measurement error will be obtained by the difference between the actual distance and the calculated distance between transmitter and detector.

## 5. Determination of the Multipath Effect

This section shows the process carried out to calculate the effects of MP when using AoA and PoA. Some example results will be shown for a tuple of emitter and detector locations, which will be conveniently enlarged in the results section.

### 5.1. Procedure for Determining the Multipath Effects in Angle of Arrival-Based Positioning

To understand the process of calculating the MP affectation error, all the steps that have been necessary to reach the final result are shown next. First, we will obtain the signal strength of the different MP effects that arrive at the PSD with 1, 2, and 3 rebounds (the reflected signal strength after 3 rebounds is considered to be very weak). The algorithm described in [46] has a setup of a particular synthetic environment, providing the feature parameters, set-up and measurement conditions. Considering a PSD lens of 4.5 mm focal length, R1 location of the sensor (a corner in the room), and the E1 location of the emitter (Table 4), the following results are obtained.

**Figure 12:**
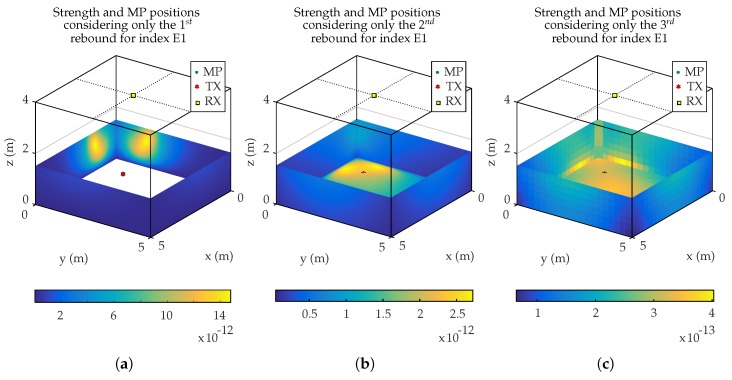
Strength and MP positions for index E1 considering only the (**a**) 1st, (**b**) 2nd, and (**c**) 3rd rebound.

Figure 12 shows the signal strength received on the PSD from each cell in which the environment is divided, considering 1 rebound (Figure 12a), 2 rebounds (Figure 12b), and 3 rebounds (Figure 12c). Each point in the figure has a different color depending on the signal strength captured by the PSD from the cell at the same point. It should be noted that the signal power shown in the figure correspond to the last rebound considered after calculating the previous ones. Let us analyze the simulation considering 2 rebounds. First of all, we calculate the emitter signal strength that is received by all cells in the environment. Next, we obtain, from each of these cells, the signal strength that reaches all the others. Finally, the algorithm obtains the signal strength that reaches the PSD from these cells, as it is shown in Figure 12b.

**Figure 13:**
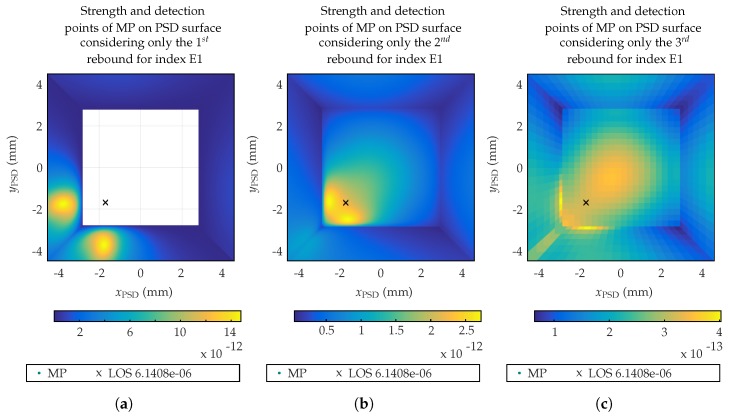
Strength and detection points of MP on PSD surface considering only the (**a**) 1st, (**b**) 2nd, and (**c**) 3rd rebound.

Figure 13 shows the signal strength and detection points of MP on PSD surface of 9×9 mm^2^, considering only the 1st rebound (Figure 13a), 2nd rebound (Figure 13b), and 3rd rebound (Figure 13c).

White area in Figure 12a and Figure 13a is due to the fact that there is no signal coming from the floor surface for *k* = 1 rebounds. The point (marked with an ‘x’) shows the impact of the LOS component, the location of the emitter. The received signal strength values shown in the legend of previous figures are normalized values (W/W) with respect to the emitted signal strength, having a linear behavior with the signal strength.

**Figure 14:**
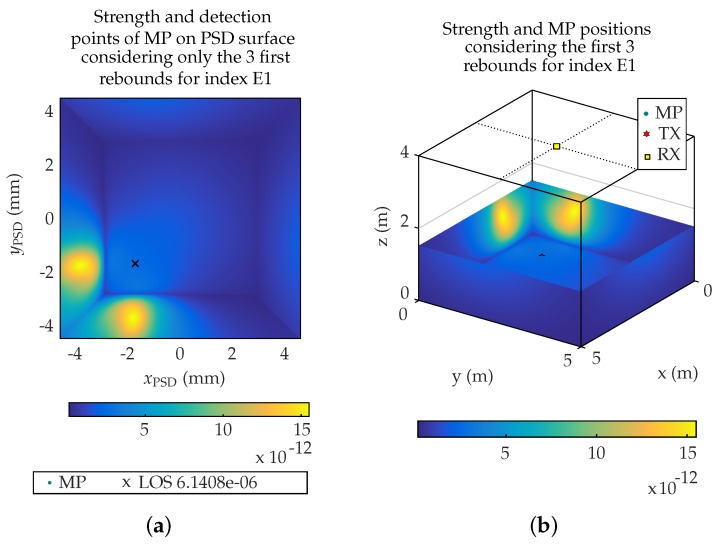
Position and strength of MP considering the first 3 rebound on (**a**) PSD surface and (**b**) in environment.

Figure 14a shows the sum of the signal strength when considering the MP effects from the first 3 rebounds on the PSD surface.

Figure 14b shows the reprojection of signal strength coming from the different cells of the environment.

To obtain the position of the emitter considering MPs, the center of mass of the received signal strength from all the MP and LOS signals is calculated and next this point is reprojected on the floor plane where the emitter moves.

**Figure 15:**
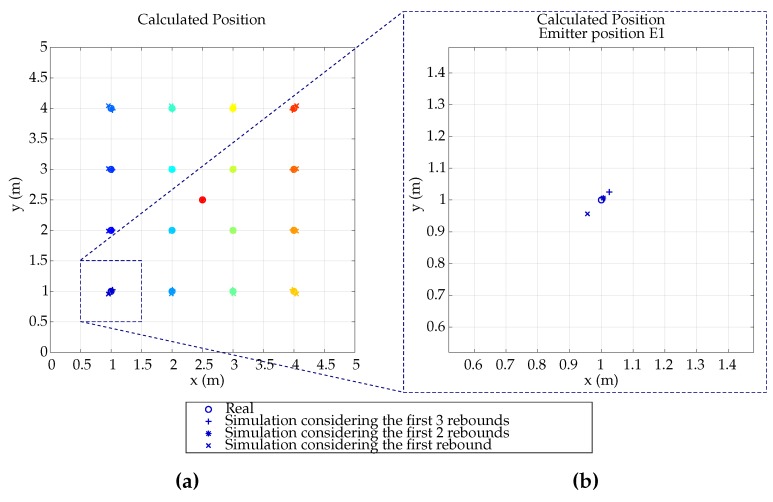
Calculated position depending on number of considered rebounds (**a**) in all emitter positions; (**b**) in emitter position of the example E1.

Figure 15 shows, for a simulation of 4 × 4 grid emitter positions equally distributed on the environment floor (Figure 15a), the calculated location using the first 3 rebounds. Figure 15b shows a zoomed area to better see the MP effects on the calculation.

It is observed that MP effect causes the calculated position of the emitter to deviate about 2 cm from the real position, despite the fact that the emitter is close to 2 walls with an important MP component and working with a 4.5 mm focal lens.

**Figure 16:**
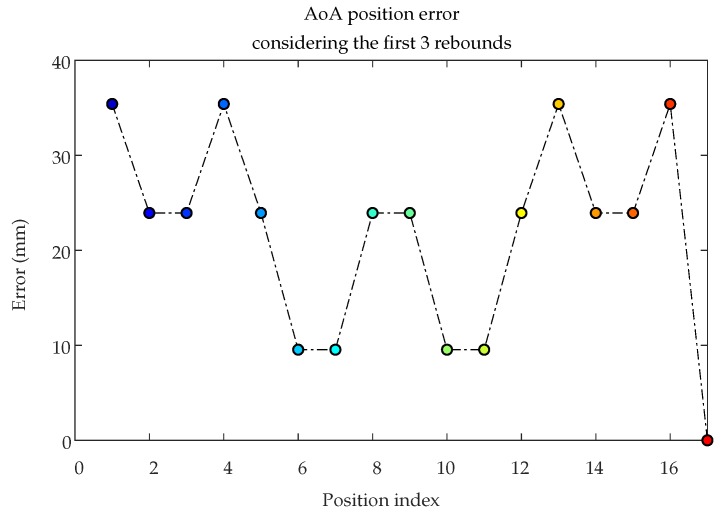
Position errors considering the first 3 rebounds.

Once the procedure for calculating the position of the emitter considering MP effects is explained, now Figure 16 shows the emitter positioning errors considering the detector fixed at point R6 while placing the emitter in the same 4 × 4 grid and including MP effects up to the first 3 rebounds.

Figure 16 shows the error depends on the position of the emitter and is greater near the corners where there are considerable rebound effects on two walls. However, at position E17 (center), the error is zero because the effects are fully compensated, due to the symmetry.

**Figure 17:**
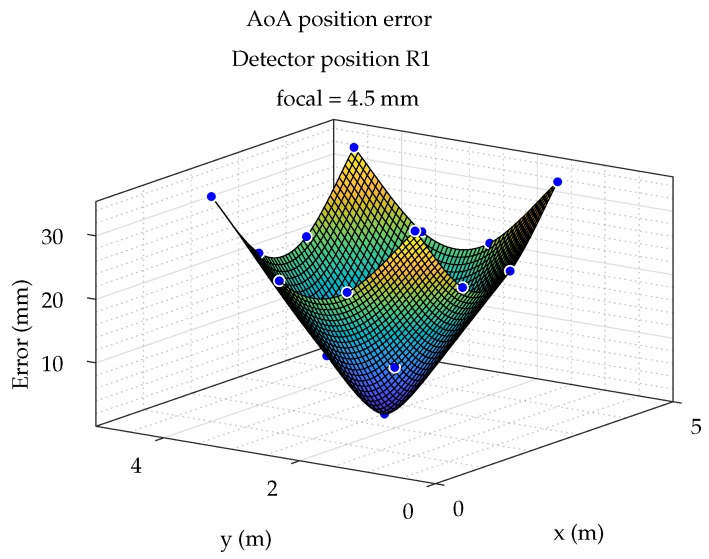
Surface of position error.

Finally, in Figure 17, the positioning errors as a function of the emitter position are shown as an error surface. For this purpose, the error values for the 17 emulated points were computed and the error surface was obtained by a thin-plate spline interpolation.

### 5.2. Procedure for Determining the Multipath Effects in Phase of Arrival-Based Positioning

Similar to the previous section, the procedure to obtain the MP effects in PoA-based positioning is shown. The same synthetic data (detector and emitter positions) will be used.

The algorithm described in [46] allows to obtain the impulse response knowing the environment features and position of emitter and detector.

It is important to note that in the PoA case we must consider both the power and phase of the different signal components reaching the detector. As the PoA technique uses the value of the signal phase, it requires knowing the impulse response of the channel.

In order to calculate the MP effects, first of all, the impulse response will be obtained for the LOS path. Additionally, impulse responses will also be obtained for signals that reach the detector after 1, 2, and 3 rebounds. Finally, the total response will be calculated.

We will use the following naming convention: $h_{0}$ is the impulse response for LOS signal; $h_{1}$, $h_{2}$ y $h_{3}$ the corresponding impulse responses for the 1st rebound, 2nd rebound, 3rd rebound, and $h_{T}$ the summation impulse response.

**Figure 18:**
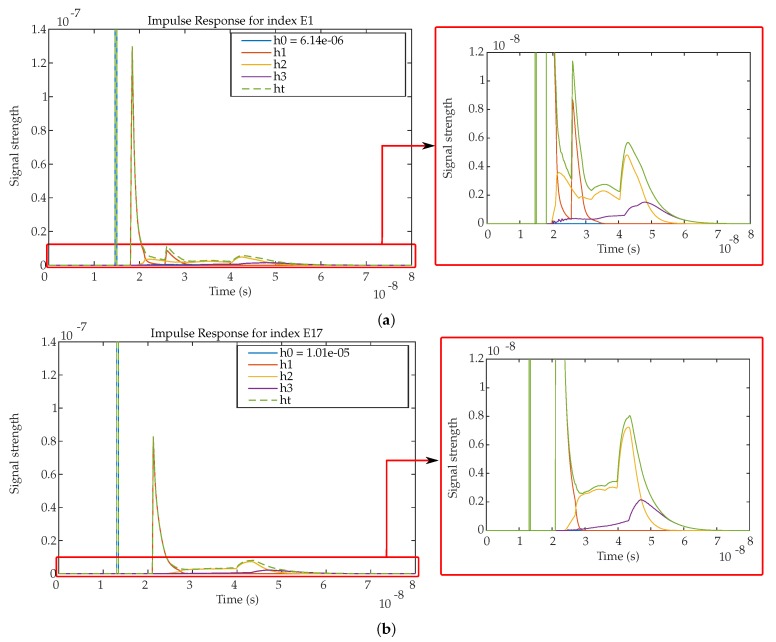
Impulse response for (**a**) index E1 and (**b**) index E17.

For example, considering the detector placed at R1 position with a focal length of 4.5 mm and the emitter positioned in the E1 and E17 positions, the impulse responses obtained are shown in Figure 18.

Each subfigure shows separately the impulse responses described before (LOS and rebound components), together with the total impulse response. It should be noted that $h_{0}$ is truncated so its peak value has been written in the legend. At the right side of each subfigure a zoomed area is shown to better quantify the MP effects from 2 and 3 signal rebounds.

It is clearly observed that $h_{0}$ of E17 arrives earlier and with more power (RSS: $1.01\times 10^{-5}$; t: $1.32\times 10^{-8}$ s) than $h_{0}$ of E1 (RSS: $6.14\times 10^{-6}$; t: $1.48\times 10^{-8}$ s), because the distance between the emitter and the detector is smaller in position E17 than in position E1. It can be also noted that $h_{1}$ of E1 has 2 significant peaks. The highest one corresponds to the MP signals reflected on the 2 closest walls and the second peak is the result of the MP signals coming from the 2 farthest walls. The impulse response $h_{1}$ of E17 only has one significant maximum, because the emitter and detector are right in the middle of the room. The maximum corresponds to the MP signals reflected from the 4 walls (identical travel distances). Also it can be seen in both cases that $h_{2}$ and $h_{3}$ have their maximum after $h_{1}$ because MPs have to travel a longer path to reach the detector.

Once the response to the impulse of the channel is known, the distance between the emitter and the detector can be calculated using the Equation (20).

**Figure 19:**
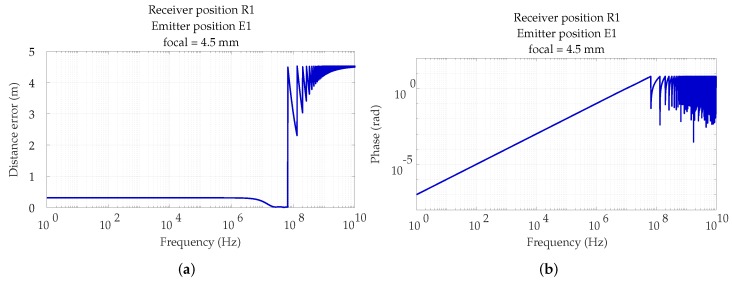
(**a**) Distance error vs Frequency; (**b**) Phase vs Frequency.

As it was mentioned above, the frequency of the simulated sinusoidal signal is 50 kHz. It should be noted that it is a very low frequency to obtain accurate phase measurements in real PoA systems, because clock errors, synchronization, and other disturbances would introduce high phase measurement errors. However, since this paper is only intended to analyze the effects of MPs, we will use the same detector features in the simulation. However, the values shown below are valid for any photodetector that may work more frequently. Figure 19a analyzes how frequency influences distance measurement errors, due only to MP errors. The distance measurement error for different frequencies depends on the environment, emitted signal, and phase calculation technique used.

Figure 19b shows the phase measurements for each frequency value obtained from the impulse response.

In Figure 19 we can see how distance errors, which are measured from the phase shift, have 3 differentiated zones. In the first zone, located from 1 to approximately $5\times 10^{6}$ Hz, the error remains constant. From this last frequency up to approximately $8\times 10^{7}$ Hz, the error decreases. This is because the distance between the emitter and detector is still below the wavelength of those frequencies. However, the indirect paths arrive at the detector with delays greater than 2π. So the detector, receiving sinusoidal signals, is not able to detect that the phase shift is greater than 2π. The third zone is located from $8\times 10^{7}$ Hz where the distance between emitter and receiver is greater than the wavelength and, therefore, the detector can not know what is exactly the emitter position. We can also see that our receiver would have to be able to measure a phase shift of 5 mrad, for the chosen frequency of 50 kHz. However, if another photodetector was chosen that could work with 5 MHz sinusoidal signal, the measured phase shift would be 0.5 rad.

**Figure 20:**
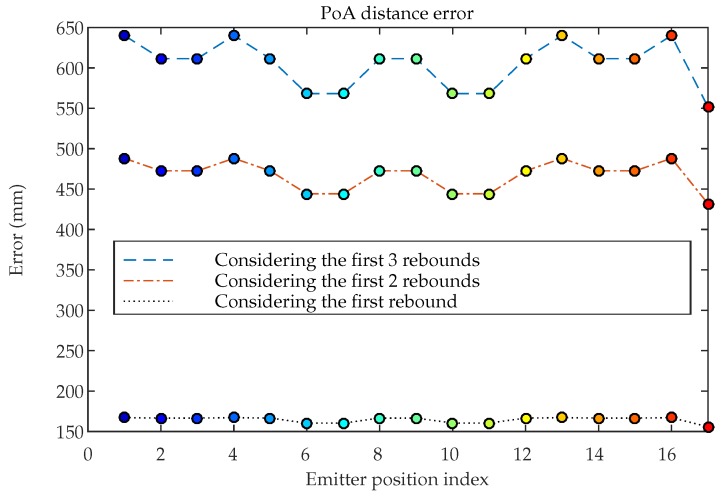
Distance error considering the first, the first 2, and the first 3 rebounds.

Figure 20 shows the errors in the distance measurement calculated from the phase of the LOS and non-LOS signals considering the 17 emitter positions. Position index of the abscissa axis corresponds to the positions $E_{i}$ indicated in Figure 3.

In this case, the distance errors shown have been obtained considering only 1 rebound, the first 2 rebounds, and 3 rebounds, to show the importance of the phase of the signals coming from third rebound signals. Although the signal strength of the MP decreases significantly with the number of rebounds, the phase offset with respect to the LOS signal introduces significant errors in the phase calculation.

Similar to AoA case, considering more rebounds does not mean significant differences in the simulated results since, although the phase offset is greater, the signal power is so small that it does not significantly affect the phase calculation.

The error is significant not only in areas close to walls (a fact that already confirmed empirically), but that it remains fairly constant throughout the space, generating errors up to 65 cm in measurements of distance between 4 and 6 m.

**Figure 21:**
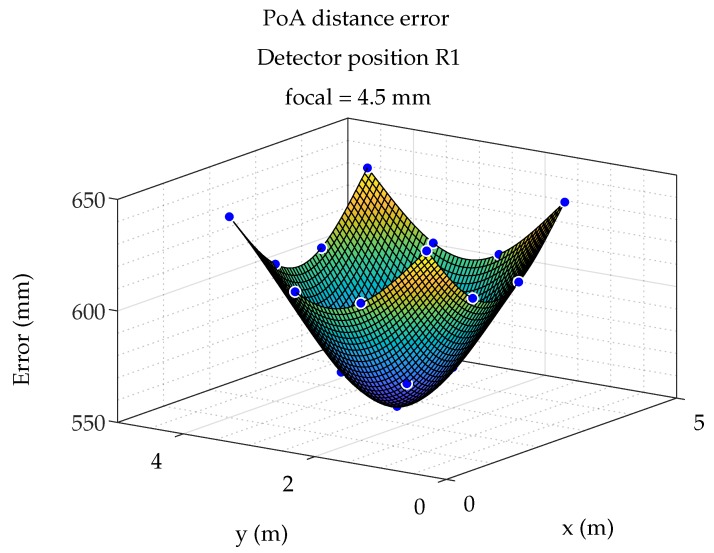
Surface of distance error.

Similar to the AoA case, Figure 21 shows the distance measurement errors as an interpolated surface considering the detector placed in the R1 position and the emitter in the 17 simulated positions.

In the next section, Results, PoA errors obtained for different detector positions are obtained and compared with the values obtained by AoA technique considering identical test conditions.

## 6. Results

This section presents the errors obtained with the detector located using AoA, and distance between emitter and receiver using PoA, in different positions and moving the emitter throughout the space, also we present results from some empirical tests. The errors shown are those that would occur due to MP effects only.

Conditions to carry on the emulated tests are the same as those depicted in Section 2.1. Therefore, the different $R_{i}$ positions for the detector and $E_{i}$ locations for the emitter are shown in Figure 3 and Figure 7. The focal lengths used in tests are 4.5 mm, 7.5 mm, and 16 mm.

The first test case has a large FoV (coverage area) and the MP effects coming from the walls will be more significant than the information coming from the emitter moving on the floor.

The 7.5 mm focal length covers a large area of displacement and partially the walls (the coverage of part of the walls only provides MP noise). The 16 mm focal length partially covers the agent’s area of motion; this is particularly important because, considering a single lens optical system, given the dimensions of the PSD sensor, the minimum focal length commercially available is 16 mm. To use a smaller focal length we would have had to resort to lens groups, but these would largely degrade the SNR. The empirical tests have been carried out with a focal length of 16 mm, indicating the conditions of realization, and when necessary emulating the tests in the same conditions.

The tests carried out show that the initial hypothesis that MP effects for AoA measurements is much less significant than for PoA technique is correct and that the errors obtained from AoA technique have little influence in the measurement.

In addition, to verify the validity of the hypothesis and the work, empirical tests will be carried out to demonstrate that the proposed models and results obtained are valid.

### 6.1. Comparison of Emulation Results

**Figure 22:**
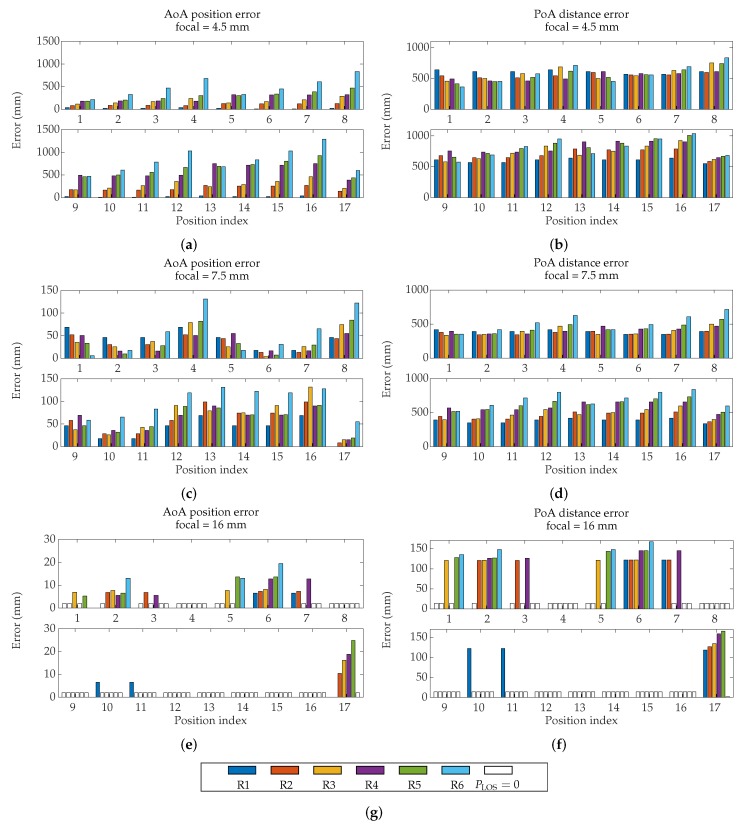
Position error of all combination of Ei and Ri using focal length of (**a**) 4.5 mm, (**c**) 7.5 mm, and (**e**) 16 mm. Distance error of all combination of Ei and Ri using focal length of (**b**) 4.5 mm, (**d**) 7.5 mm, and (**f**) 16 mm. (**g**) Shows bar color identifier for each receiver position.

As a result of the procedures described before, the measuring system has been emulated with the detector in every locations $R_{i}$, and emitter in $E_{i}$ positions. Figure 22 show the results obtained using AoA and PoA respectively with focal lengths of 4.5 mm, 7.5 mm, and 16 mm, for each combination of $E_{i}$ and $R_{i}$ positions.

The errors obtained by PoA are much more significant than those obtained by AoA. Additionally, the PoA errors have similar behavior no matter the different positions of the detector; this indicates that the MP by PoA cannot be mitigated with strategic placement of detectors in the environment. The MP errors in AoA are a magnitude lower.

It is worth noting that AoA calculates the emitter position while PoA obtains only the distance between emitter and detector (considering one single PSD). The two measurements are therefore not directly comparable, but considering that position from PoA is possible if multiple detectors are used, the errors shown here will be maintained or increased [71], therefore we can draw sufficient conclusions in order to state which technique is most influenced by MP effects.

They depend strongly on the position of the detector, so by choosing a convenient location the effects of the MP can be greatly mitigated. As our hypothesis indicated, when the MP signal reaches the whole surface of the PSD detector, it causes “MP noise” current to be generated in each electrode and its effects to be counteracted. So, the more symmetrical the space around the detector is, the less important the multipath effects are.

Another very remarkable aspect is that in both techniques the value of the focal length is very relevant. The results are greatly improved if the procedure used is AoA. When a small focal length is available, the detector has a FoV greater than the emitter’s movement plane, and the MP signals comes from these reflection areas. Therefore, the smaller the part of the FoV outside the displacement zone, the less the MP will affect. This is more significant when, measuring by AoA, we only capture the emitters movement zone and not walls (focal lengths of 16 mm and 7.5 mm in R1, R2, and R3 detector positions) giving MP errors fairly constant depending solely on the position of the emitter and with a very low error (less than 2 cm with f=16 mm).

### 6.2. Error Curves as a Function of the Focal Length and Position of the Detector

**Figure 23:**
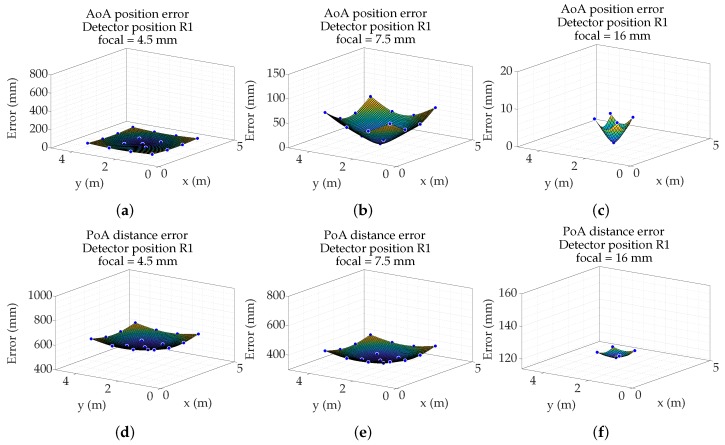
Surface of Angle of Arrival (AoA) position error in R1 using focal length of (**a**) 4.5 mm, (**b**) 7.5 mm, and (**c**) 16 mm; and surface of PoA distance error in R1 using focal length of (**d**) 4.5 mm, (**e**) 7.5 mm, and (**f**) 16 mm.

**Figure 24:**
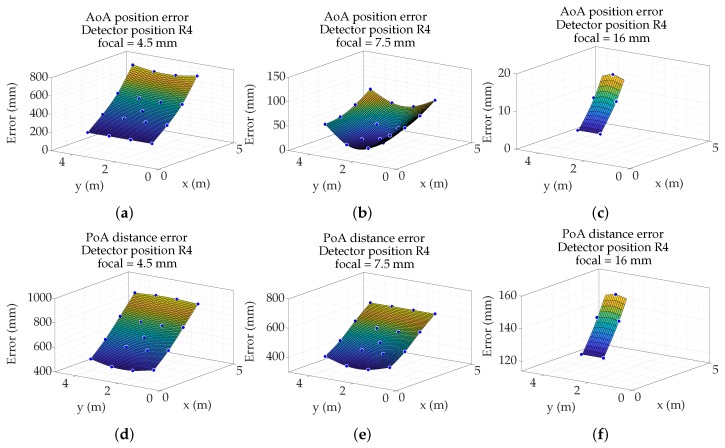
Surface of AoA position error in R4 using focal length of (**a**) 4.5 mm, (**b**) 7.5 mm, and (**c**) 16 mm; and surface of PoA distance error in R1 using focal length of (**d**) 4.5 mm, (**e**) 7.5 mm, and (**f**) 16 mm.

From the results shown in Figure 22, a thin-plate spline interpolation has been performed to obtain the error curves for each focal length and each detector location in the entire movement space of the emitter. Figure 23 and Figure 24 show, as an example, results on two detector positions.

After the previous tests, we have proceeded to emulate which would be the errors due to the MP effects in the demonstrators of our research group. In this case it is a corridor 3.5 m high, 3.5 m wide, and 20 m long. We have used a PSD S5991-01 from Hamamatsu Photonics (Hamamatsu, Japan) with $9\times 9\;{\mathrm{mm}}^{2}$ area, assembly with a 16 mm focal length lens.

An important aspect to deal with is that, given the length of the corridor, several sensors must be deployable in order to achieve complete coverage. Here it is important to select a suitable optic, which, given the results obtained in the previous section, should be greater than 7.5 mm in order not to pick up MP noise from the walls. However, the lens used for the empirical tests will be 16 mm focal length; this is imposed because our sensor is the Hamamatsu PSD S5991-01 9×9 ${\mathrm{mm}}^{2}$, so the diameter of the lens has to be 25.4 mm (1 inch) and the minimum focal length marketed is 16 mm for single lenses. For shorter distances a group of lenses should be fitted, which would introduce a loss of SNR

**Figure 25:**
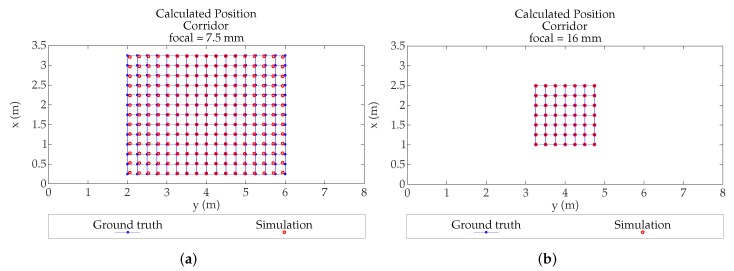
Calculated position in an emulated corridor (**a**) using focal length of 7.5 mm; (**b**) using focal length of 16 mm.

Figure 25a,b show the calculated position of an agent in the corridor (with 7.5 mm and 16 mm focal length, respectively). In both cases the detector is placed centered on the ceiling. The points showed in figures are only those that have coverage of the detector.

**Figure 26:**
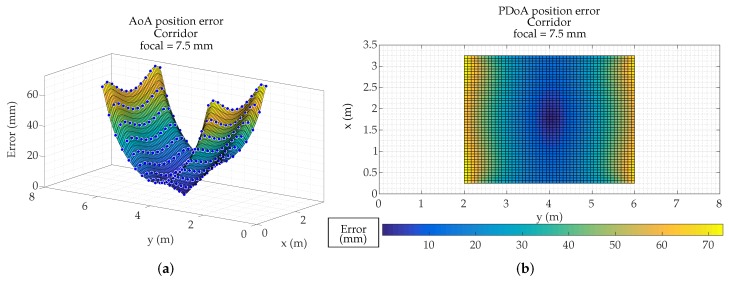
Position error in an emulated corridor using focal length of 7.5 mm. (**a**) 3D representation; (**b**) 2D colormap representation.

**Figure 27:**
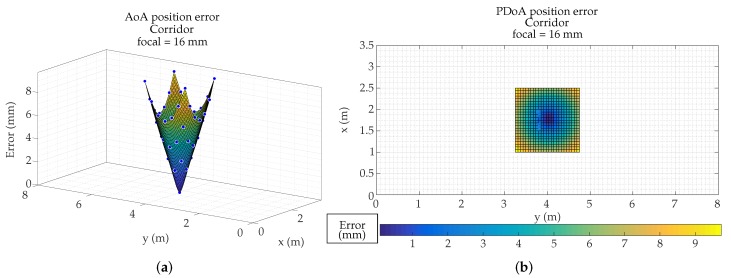
Position error in an emulated corridor using focal length of 16 mm. (**a**) 3D representation; (**b**) 2D colormap representation.

Figure 26 and Figure 27 show the MP errors, to position an agent in the corridor (with 7.5 mm and 16 mm focal length respectively). MP errors have been obtained from calculated position shown in Figure 25. The points color indicates the error that would be made in obtaining the location of an emitter on that point. It should be noted that the scale of Figure 26 and Figure 27 are different in order to observe in more detail the error as a function of the emitter position.

The focal length case of 7.5 mm has a larger coverage, but is also more sensitive to multipath effects.

In the case of the 16 mm lens, it does not cover the entire width of the corridor and has areas free of coverage. As can be seen the maximum errors with 7.5 mm lenses and in the areas farthest from the detection would be 6 cm. In the case of the 16 mm focal, the coverage does not reach the width of the corridor, since it only covers 1.5 m (it would be greater if it could be placed higher), but, nevertheless, when the agent passes through the coverage area, the errors due to multipath would be a few millimeters in the whole area. This has a positive reading, as it means that the system could be placed in a corridor up to 1.5 m wide without being affected by the multipath.

### 6.3. Comparative Results of Emulation and Empirical Tests

**Figure 28:**
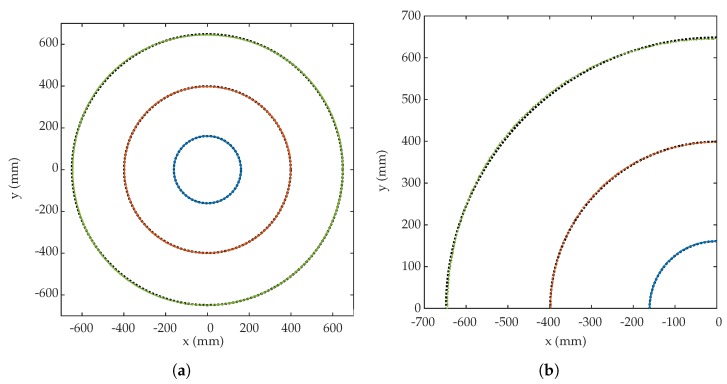
(**a**) Measured (color dots) and best fit circle (black dot line) of empirical tests with radius 160 (blue), 400 (red), and 650 mm (green). (**b**) Zoom version of (**a**).

In order to be able to compare the results, several tests have been carried out on a scenario such as the one simulated in the previous section with a 16 mm lens. Two tests have been carried out; in the first one, several circles path are described with radius 650 ± 2 mm, 400 ± 2 mm, 160 ± 2 mm. The results shown in Table 5 and in Figure 28 have been obtained.

In the second test, in the same area as the previous test, circles with a radius of 725 ± 2 mm are described, in this case a fixed wall of highly reflective foam is placed on at a distance of 10 cm of the sides of the circles drawn by the emitter, in order to create MP effects.

**Figure 29:**
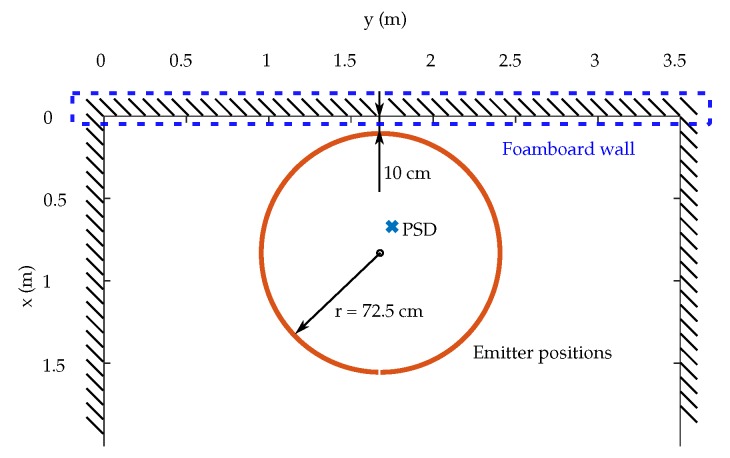
Environment of fixed wall test.

In Figure 29 is shown position of PSD sensor and emitter in the environment.

**Figure 30:**
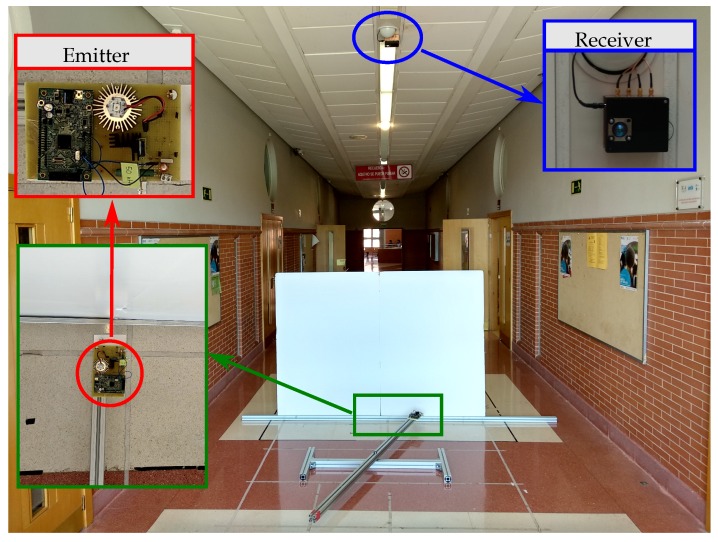
Setup of the real scenario.

The setup of the real scenario is shown in Figure 30.

**Figure 31:**
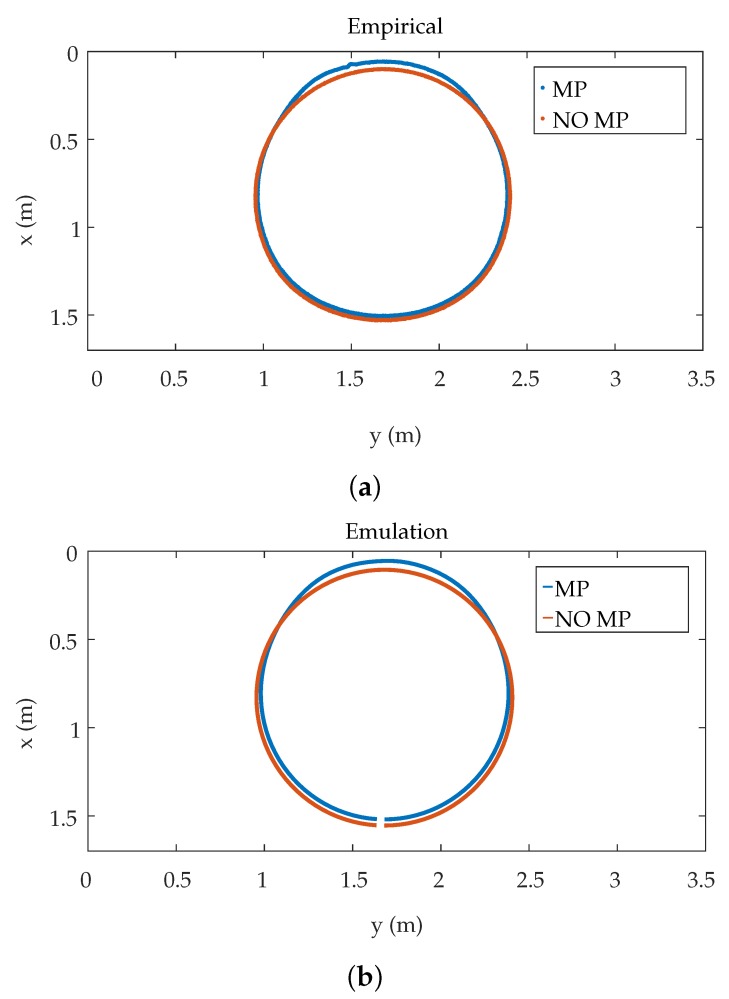
Second test (**a**) empirical results and (**b**) emulated results.

In addition to carrying out this second test, to be able to compare the concrete situation the behavior was emulated with the same model used previously. Figure 31 shows the results obtained. Figure 31a shows in red results of the empirical tests without placing the sidewall and in blue the results obtained when placing it. Figure 31b shows in red the results of the emulation without the wall and in blue the results of the emulation by placing the wall. It can be seen that both real and emulated tests have the same shape, although there is a small difference in values due to the effects of the actual tests and behaviors. In any case, it can be observed and concluded that MP errors do not affect the accuracy.

## 7. Conclusions

In this work we have characterized the positioning measurement errors that would be caused by the effects of multipath working with IR signals. The positioning has been determined and calculated using AoA and PoA techniques. It has been found, as our hypothesis indicated, that AoA is affected by an order of magnitude lower than PoA, working in both cases with a PSD photodiode. This is because in the determination of the impact point using AoA the different MPs compensate its effects. In addition, errors vary greatly with the position of the detector, indicating that sensor deployment strategies can be chosen to mitigate the MP effects. However, in PoA technique, phase shift errors are not compensated and not balanced by the same factor.

In addition, it should be noted that the MP effects on PoA are quite homogeneous, regardless of the position and orientation of the receiver.

Another important aspect is focal length of the optical lens in the detector. In narrow areas or close to walls it is advisable to work with lenses with a larger focal length, in order to avoid that the coverage of the detector covers a large area of walls, since only “MP noise” has been collected from them. In short, MP can be avoided if the coverage area does not cover wall.

The empirical tests carried out have validated the expected results from the emulations, and as shown in the last test, with a 16 mm lenses, the positioning errors in a corridor are in the range of millimeters.

## Figures and Tables

**Table 1:** Empirical surface reflection parameters for different materials.

Material	$u_{\mathrm{as}}$	$v_{\mathrm{as}}$	$u_{\mathrm{nd}}$	$v_{\mathrm{nd}}$	$u_{\mathrm{ns}}$	$v_{\mathrm{ns}}$	K
Terrazzo (floor)	$1.713\times 10^{-2}$	−2.379	1.021	0.428	34.55	−0.377	0.414
Plaster board (ceiling)	$0.128\times 10^{-2}$	−4.911	1.083	0.463	33.41	1.382	0.521
Foam board (walls)	$1.742\times 10^{-2}$	−3.103	1.457	1.005	102.1	0.731	0.388

**Table 2:** Cell size and number of indirect paths depending on the number of rebounds.

Rebound	Cell Size	Number of Indirect Paths (MP)
1st	$1\times 1\;{\mathrm{cm}}^{2}$	$1.3\times 10^{6}$
2nd	$5\times 5\;{\mathrm{cm}}^{2}$	$2.7\times 10^{9}$
3rd	$25\times 25\;{\mathrm{cm}}^{2}$	$9\times 10^{9}$

**Table 3:** Receiver orientation depending on its position and focal length.

Detector	f=4.5mm			f=7.5mm			f=16mm		
Position	x	y	z	x	y	z	x	y	z
R1	0	0	−1	0	0	−1	0	0	−1
R2	0	0	−1	0.184	0	−0.983	0	0	−1
R3	0	0	−1	0.170	0.170	−0.971	0	0	−1
R4	0	0	−1	0.391	0	−0.920	0.160	0	−0.987
R5	0	0	−1	0.385	0.170	−0.907	0.160	0	−0.987
R6	0	0	−1	0.348	0.348	−0.870	0.158	0.158	−0.974

**Table 4:** Focal length of the lens, receiver and emitter location used in the test.

Focal Length of the Lens	Receiver Location	Emitter Location
4.5 mm	R1	E1

**Table 5:** Empirical results of the first test.

Ground Truth Radius (mm)	Measured Radius (mm)	Circle Adjustment (mm)		
		*STD*	*RMSE*	*Error Max*
650 ± 2	648.23	0.10	0.05	1.88
400 ± 2	398.91	0.11	0.03	2.29
160 ± 2	159.87	0.08	0.02	1.21

## Abbreviations

The following abbreviations are used in this manuscript:

AoA	Angle of Arrival
FoV	Field of View
IPS	Indoor Positioning System
IR	Infrared
LOS	Line of Sight
LPS	Local Positioning System
MP	Multipath
PoA	Phase of Arrival
PSD	Position Sensitive Device
